# UHPLC-MS/MS-Based Metabolomics and Clinical Phenotypes Analysis Reveal Broad-Scale Perturbations in Early Pregnancy Related to Gestational Diabetes Mellitus

**DOI:** 10.1155/2022/4231031

**Published:** 2022-08-24

**Authors:** Ting Hu, Zhuoling An, Han Li, Yanping Liu, Liangyu Xia, Ling Qiu, Aimin Yao, Liangkun Ma, Lihong Liu

**Affiliations:** ^1^Beijing Chao-Yang Hospital, Capital Medical University, Beijing 100020, China; ^2^Peking Union Medical College Hospital, China Academic Medical Science and Peking Union Medical College, Beijing 100730, China; ^3^Shunyi District Maternal and Child Health Hospital, Beijing 101320, China

## Abstract

Gestational diabetes mellitus (GDM) is the most common metabolic disturbance during pregnancy, with adverse effects on both mother and fetus. The establishment of early diagnosis and risk assessment model is of great significance for preventing and reducing adverse outcomes of GDM. In this study, the broad-scale perturbations related to GDM were explored through the integration analysis of metabolic and clinical phenotypes. Maternal serum samples from the first trimester were collected for targeted metabolomics analysis by using ultra-high performance liquid chromatography-tandem mass spectrometry (UHPLC-MS/MS). Statistical analysis was conducted based on the levels of the 184 metabolites and 76 clinical indicators from GDM women (*n* =60) and matched healthy controls (*n* =90). Metabolomics analysis revealed the down-regulation of fatty acid oxidation in the first trimester of GDM women, which was supposed to be related to the low serum level of dehydroepiandrosterone.While the significantly altered clinical phenotypes were mainly related to the increased risk of cardiovascular disease, abnormal iron metabolism, and inflammation. A phenotype panel established from the significantly changed serum indicators can be used for the early prediction of GDM, with the area under the receiver-operating characteristic curve (ROC) 0.83. High serum uric acid and C-reaction protein levels were risk factors for GDM independent of body mass indexes, with ORs 4.76 (95% CI: 2.08-10.90) and 3.10 (95% CI: 1.38-6.96), respectively. Predictive phenotype panel of GDM, together with the risk factors of GDM, will provide novel perspectives for the early clinical warning and diagnosis of GDM.

## 1. Introduction

Glucose metabolism during pregnancy differs from that of non-pregnancy because pregnant women must meet the energy needs of both themselves and the embryo [1–3]. Therefore, gestational women are usually associated with insulin resistance and hyperinsulinemia, which can lead to diabetes in some expectant mothers [1, 4]. Gestational diabetes mellitus (GDM) is defined as varying degrees of glucose intolerance that develops or is first detected during pregnancy [5]. Risk factors of GDM include overweight, high-fat diet, high-sugar diet, micronutrient deficiencies, advanced gestational age, and family history of diabetes [6, 7]. In recent decades, with the improvement of quality of life, the incidence of GDM in China has been on the rise. The high prevalence of GDM is of great concern because gestational women suffering from GDM have a much higher risk of type 2 diabetes, cardiovascular diseases (CVD), and female malignancies after delivery compared to the healthy controls [5, 7]. About 15-45% of the offspring of GDM mothers are macrosomia, which is a 3-fold higher rate compared to normal controls [8]. In addition, GDM can also cause abnormal development of the embryo and increase the rate of miscarriage [9, 10].

The pathogenesis of GDM is not fully understood. The maternal-fetal and lifestyle factors are interrelated and acted in an integrated manner in the development of GDM. A study of healthy, thin women showed a 56% decrease in insulin sensitivity and a 30% increase in basal endogenous glucose production in the third trimester compared to pre-pregnancy [5, 11, 12]. Insulin resistance develops around the second trimester and progresses throughout the rest of the pregnancy [5]. GDM is usually the result of *β*-cell dysfunction on a background of chronic insulin resistance during pregnancy [13]. Increased steroid hormones like estrogen, progesterone, and cortisol during pregnancy can also contribute to the disruption of balance existed between glucose and insulin. In addition, unhealthy lifestyles and high caloric intake are also important factors for the occurrence of GDM [8].

Screening for GDM is usually performed between 24 and 28 weeks' gestation using the 75 g oral glucose tolerance test (OGTT). A “one-step” 2 hours 75 g OGTT is endorsed by the International Association of Diabetes and Pregnancy Study Groups (IADPSG) and World Health Organization (WHO) [5]. Lifestyle interventions and occasional insulin therapy are currently the only strategies to prevent or treat GDM, but their effect is limited due to insulin resistance. The risk assessment of diabetes in the first trimester is of great significance for the prevention of GDM. As the levels of endogenous metabolites are usually influenced by both genetic and environmental factors, metabolomics analysis has proved to be an important method to elucidate metabolic changes underlying the pathogenesis of GDM and lead to the discovery of potential diagnostic biomarkers for GDM [14–16]. However, current studies mostly focused on the variation of metabolite concentrations and the disturbance of metabolite pathway, while ignored the clinical phenotypes [14, 17]. This is not conducive to comprehensively elucidate the physiological function of the significantly changed metabolites and explore the actual pathogenesis of GDM.

Considering the adverse effects of maternal hyperglycemia on the mothers and their offspring, this study investigated the metabolic and clinical phenotypic disturbances associated with GDM in early pregnancy. A targeted metabolomic study was performed using ultra-high performance liquid chromatography-tandem mass spectrometry (UHPLC-MS/MS) on serum samples from 60 GDM mothers and 90 controls in the first trimester. A total of 184 metabolites were detected in the serum samples. Integration analysis was conducted based on the levels of the 184 metabolites and 76 clinical indicators to explore early diagnostic biomarkers and risk factors for GDM.

## 2. Experimental Section

### 2.1. Chemicals and Reagents

The commercial metabolites standards were purchased from Cayman Chemical (Ann Arbor, MI, USA), Bidepharm (Shanghai, China), Sigma-Aldrich (St. Louis, MO, USA), or Steraloids (Newport, RI, USA). Eight isotopic internal standards (ISs), including thymine-*d4*, valine-*d8*, phenylalanine-*d8*, 17-hydroxyprogesterone-*d8*, docosahexaenoic acid-*d5*, cholic acid-*d4*, chenodeoxycholic acid-*d4*, and glycocholic acid-*d4*, were obtained from Cambridge Isotope Laboratories (Cambridge, MA, USA). Ultrapure water was prepared by a Milli-Q purification system (Bedford, MA, USA). HPLC or MS grade solvents, including acetonitrile, methanol, isopropyl alcohol, and formic acid, were purchased from Fisher Scientific (Pittsburgh, PA, USA).

### 2.2. Ethical Statement

This study was approved by the ethics committees of Peking Union Medical College Hospital of the Chinese Academy of Medical Science. The study was registered on http://www.ClinicalTrials.gov (registration ID: NCT03651934) and conducted in accordance with the Declaration of Helsinki. All the participants received details of the study and signed consent forms. The participants were free to withdraw from the study at any time.

### 2.3. Study Population

An aliquot of 2 mL fasting blood sample was collected from each of 414 pregnant women who attended their first pregnancy test at the outpatient of the Shunyi District Maternal and Child Health Hospital (Beijing, China) from October to December 2018. The blood was centrifuged at 3000 g for 10 min, and the upper serum was collected. All samples were stored at -70°C for subsequent analysis. Later, a face-to-face interview was conducted to collect basic demographic information of the participants, including exercise contraindication, food allergy history, medication history, previous pregnancy history, and family disease history. Clinical chemistry parameters at blood collection time points were collected from the hospital medical record system. The 75 g OGTT was conducted for the diagnosis of GDM between 24^th^ and 28^th^ gestational weeks according to the criteria of IADPSG [5]. GDM can be diagnosed if any of the following conditions of OGTT were obtained: fasting plasma glucose ≥5.1 mmol/L, 1 h glucose level ≥10 mmol/L, or 2 h glucose level ≥8.5 mmol/L. [5] The information on Infant birth weight and body length was recorded.

**Figure 1:**
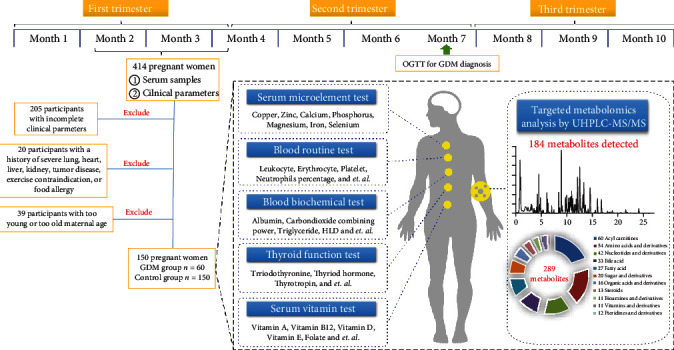
Participant flow chart and research schematic.

All the serum samples from 414 participants were applied for metabolomics analysis. In a subsequent statistical analysis, 205 participants with incomplete clinical indicators were excluded. Another 20 participants with a history of severe lung, heart, liver, kidney, or tumor disease were excluded. Thirty-nine participants with too young or too old embryonic age were excluded. Finally, data from 150 participants with embryonic age between 6 and 14 weeks were included in the final statistical analysis, including 60 GDM cases and 90 healthy controls. A flow chart of the inclusion and exclusion of the participants is shown in Figure 1.

### 2.4. Targeted Metabolomics Analysis by UHPLC-MS/MS

Targeted metabolomics analysis was conducted by reverse phase ultra-high performance liquid chromatography coupled with tandem mass spectrometry. A Spark Holland liquid chromatography system (Spark, Holland) coupled with an API 5500 mass spectrometer (AB Sciex, Canada) with an electrospray ionization (ESI) source was employed for the targeted metabolomics analysis. Chromatographic separation was performed on a HSS T3 (150 × 2.1 mm, 3.5 *μ*m) column. Protein precipitation method was used for biological sample preparation. Each 50 *μ*L serum sample was mixed with 10 *μ*L ISs solution and 140 *μ*L cold methanol (-20°C). The mixture was vortexed for 2 min and then centrifuged for 10 min at 4°C. The supernatants were collected and directly injected to the liquid chromatography for metabolomics analysis.

All metabolites and isotope ISs were analyzed in a single injection using both negative and positive modes with rapid polarity switching (50 ms) and advanced scheduled multiple reaction monitoring (s-MRM) algorithm. More detailed parameters regarding the methods were published in our previous report [18]. The pooled serum mixed by equal aliquot of serum samples from dozens of healthy volunteers was used as quality control (QC) samples during metabolomics analysis. For data quality assessment during batch analysis, pooled QC samples were processed as real samples and inserted into the analytical runs every 12 samples. The MRM parameters were listed in Supplementary Table S1.

### 2.5. Data Processing and Statistical Analysis

Raw data files from the UHPLC-MS/MS analysis were processed using MultiQuant software (version 3.0.2, AB SCIEX). All peak areas were divided by their corresponding IS peak areas, and the ratios were plotted against the real concentrations to construct calibration curves by the least squares method with a 1/*x*^2^ weighting factor. The absolute concentration of each metabolite was calculated according to the calibration curve. For some fatty acids and acyl carnitines without standards, their relative concentrations were calculated according to the peak area ratio of an analyte to the corresponding IS. SIMCA 14.1 (Umetrics AB, Umeå, Sweden) was employed for multivariate statistical analysis, including principle component analysis (PCA) and orthogonal partial least squares discriminant analysis (OPLS-DA). The quality of each OPLS-DA model was evaluated using R2(cum) values, which identify the variations described by all components in the model. Q2(cum) was a value calculated from sevenfold cross-validation, which represents the predictability of the modeling [19]. Besides, the permutation test was used to investigate whether the OPLS-DA model was overfitted. The variable importance in projection (VIP) is the most important parameter for the evaluation of each variable in the OPLS-DA model, with VIP>1 considered to be an important contribution to the classification model. IBM SPSS 21 (Armonk, New York, United States) was used for *t*-test, Spearman's correlation, and binary logistic regression analysis (backward stepwise: Wald), with *P* < 0.05 considered statistically significant. The open source tool of Metaboanalyst (HYPERLINK: https://www.metaboanalyst.ca/) was also employed for calculating of false discovery rate (FDR). The correlation networks of metabolic and clinical phenotypes were generated by Cytoscape 3.5.0. The correlation coefficients obtained from Spearman's rank correlation analysis were imported to the Cytoscape 3.5.0 to generate the correlation networks. GraphPad Prism 7 was used for receiver-operating characteristic (ROC) and histogram analysis.

## 3. Results

### 3.1. Demographic and Clinical Characteristics of the Participants

Of the 150 participants included in the final statistical analysis, 60 participants who met the diagnostic criteria for GDM were assigned to the GDM group, and 90 participants with normal glucose tolerance were assigned to the control group. The demographic and clinical characteristics of the 150 participants are listed in Table 1. There was no significant difference in the age of pregnant women between the GMD group and the control group, with average ages all about 30 years old. There was also no significant difference in the gestational age at the time point of blood sample collection. All the participants were Chinese, with more than 90% of them were the Han nationality. Significantly higher pre-pregnancy body mass index (BMI) was observed in the GDM group compared to the control group. As shown in Table 1, more than 50% of GDM women had pre-pregnancy weight above the upper limit of normal BMI (normal BMI: 18.5-24 kg/m^2^ for Chinese), while only 30% of control women had pre-pregnancy weight larger than the upper BMI limit. According to the previous literature report, overweight was a risk factor for GDM. High pre-pregnancy BMI level was associated with a higher risk of GDM [6]. Approximately 53% of the GDM women were primipara and 47% were multiparas, with no obvious difference between the GDM and control groups. Eighteen percent of the GDM cases and 10% of the control cases had a family history of diabetes, with no significant differences observed.

Offspring information, including infant gender, infant birth weight, and infant length, were recorded for these 150 participants (Table 1). No significant difference was found in infant gender and infant length. However, the infant birth weight of the GDM participants was significantly higher than the infants delivered by the control mothers. GDM mothers were more likely to deliver overweight babies.

### 3.2. Variation of Metabolic Phenotype Related to GDM

**Figure 2:**
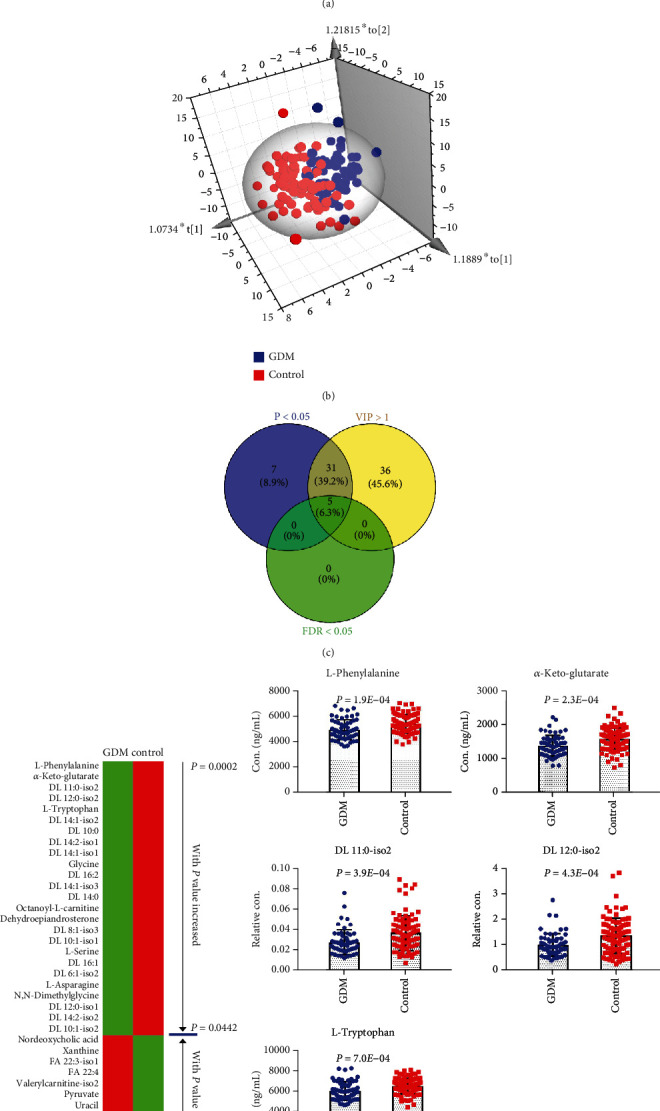
(a) Performance of QC samples. (b) OPLS-DA score plot of the GDM and control groups. (c) A Venn diagram showed that 36 metabolites were significantly changed (*P* < 0.05 and VIP>1) between GDM and control groups, with 5 of them possessed FDR<0.05. (d) Heat maps of the 36 significantly variated metabolites (*P* < 0.05 and VIP>1). (e) Histograms of the 5 metabolites with FDR<0.05.

A targeted metabolomics method established in our laboratory was employed for the metabolites profiling in serum. A total of 289 endogenous metabolites with physiological significance were covered in this targeted metabolomics method (Figure 1). Finally, 184 metabolites were detected in the serum samples of this study. Pooled serum QC samples were evenly inserted throughout the sample analytical batch to monitor the deviation introduced from sample pretreatment and instrumental analysis. A rapid systematic check of the data quality before data processing was made by performing PCA on the complete data set. As shown in Figure 2(a), 78% and 88% peaks detected in QC samples had coefficients of variation (CV) below 15% and 30%, respectively. Of the other 12% peaks with CV values in QC samples above 30%, only 7 metabolites had CV values of QC above 40%. These metabolites with large CV values were basically caused by low endogenous concentrations. Quantitative data of all the 184 metabolites detected in serum were included in subsequent statistical analysis [20, 21].

PCA was performed based on the quantification data of the 184 metabolites, and the score plot was shown in Figure S1A. The samples of the control group and the GDM group showed a slight trend of separation on the PCA score plot. The supervised OPLS-DA was further used to investigate the metabolic phenotype variation related to GDM. As shown in Figure 2(b), samples within groups were segregated into clusters in the score plot of OPLS-DA. A random permutation test with 100 iterations was performed to validate the OPLS-DA model. As shown in Figure S2, the permuted *y*-variables were all lower than the original *y*-variables. No overfitting of the OPLS-DA mode was observed. Furthermore, a bilateral *t*-test was conducted to investigate the metabolite changes related to GDM. Based on the criteria of *P* < 0.05 and VIP>1, 36 metabolites were significantly different between GDM and control groups (Figure 2(c), Table S2). PCA score plot based on the concentration levels of these 36 metabolites was shown in Figure S1B, with a more obviously separation trend between control and GDM groups than Figure S1A. The average values of the 36 metabolites in each group were presented as heat maps in Figure 2(d). Of the 36 significantly changed metabolites, a dozen of acyl carnitines were down-regulated and fatty acids were up-regulated in the first trimester of GDM gestational women compared to the normal control maternity. Finally, all the metabolites were used for FDR test. The main goal of FDR control is to set significance levels for a collection of tests in such a way that among tests declared significant; the proportion of true null hypotheses is lower than a specified threshold [22]. Five of these 36 significantly changed metabolites exhibited a FDR less than 0.05, including L-phenylalanine, *α*-keto-glutarate, DL 11 : 0-iso2 (acyl carnitine, with fatty acid carbon chain length of 11 and saturation of 0), DL 12 : 0-iso2 (acyl carnitine, with fatty acid carbon chain length of 12 and saturation of 0), and L-tryptophan. The histograms of these 5 metabolites were shown in Figure 2(e).

### 3.3. Correlation Analysis of the Significantly Changed Metabolites

**Figure 3:**
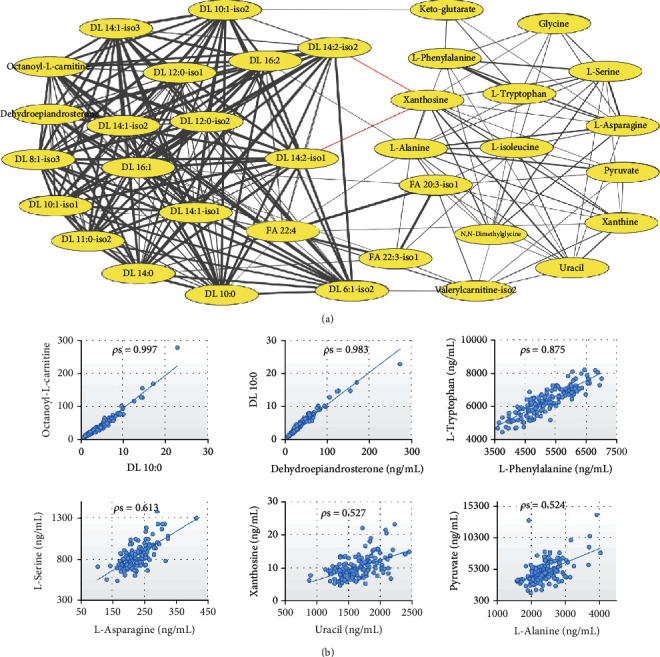
(a) Spearman's correlation (*P* < 0.01) among the significantly changed metabolites related to GDM. A gray line indicates a positive correlation and a red line indicates a negative correlation. The thicker the line, the stronger the correlation. (b) Correlation existed between some representative metabolites, with Spearman's correlation coefficients marked on the top center.

Since the endogenous metabolites present a complex network, a correlation analysis would help reveal the key nodes disturbed by the GDM pathological state. The correlations of the 36 significantly changed metabolites between GDM and control groups are shown in Figure 3(a) based on Spearman's rank correlation analysis. Only those correlations with *P* < 0.01 were shown in the figure. The majority of the metabolites presented positive correlations with each other. Due to the similarity of biosynthesis, acyl carnitines and fatty acids show strong correlations within the class. An example of the strong correlation that existed among acyl carnitines is shown in Figure 3(b), and the correlation coefficient between DL 10 : 0 (acyl carnitine, with fatty acid carbon chain length of 10 and saturation of 0) and octanoyl-L-carnitine was 0.997. It was also worth noting that dehydroepiandrosterone was strongly correlated with all acyl carnitines. As shown in Figure 3(b), the correlation coefficient between dehydroepiandrosterone and DL 10 : 0 was 0.983.

L-Phenylalanine and L-tryptophan are essential amino acids, both belonging to the aromatic amino acid. The normal metabolism of L-phenylalanine and L-tryptophan requires the participation of niacin and vitamin B6 [23–26]. A strong positive correlation was found to be existed between L-phenylalanine and L-tryptophan, with a 0.875 correlation coefficient (Figure 3(b)). They were all significantly lower in the GDM group compared to the control group (Figure 2(d)). To our knowledge, this may be the first report of the positive correlation between L-phenylalanine and L-tryptophan, suggesting that there may be some correlation between these two essential amino acids in endogenous metabolism. L-Asparagine and L-serine are nonessential amino acids that derived from oxaloacetate and L-serine, respectively. A significantly positive correlation was also found between L-asparagine and L-serine, with a 0.613 correlation coefficient (Figure 3(b)). Besides, those metabolites with clear upstream and downstream metabolic correlation, such as uracil/xanthosine and L-alanine/pyruvate, all exhibited strong positive correlations within each other (Figure 3(b)).

### 3.4. Variation of Clinical Phenotype Related to GDM

**Figure 4:**
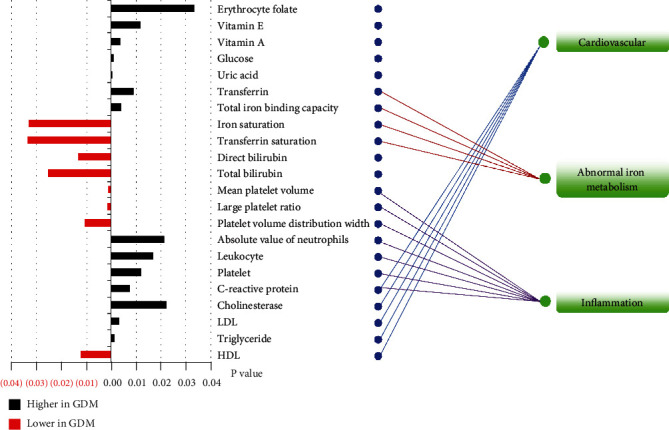
Twenty-two significantly changed (*P* < 0.05 and VIP>1) clinical phenotypes and the related disease risks related to GDM.

A total of 76 clinical phenotypes were finally included in the statistical analysis, mainly including the microelement test, blood routine test, blood biochemical test, thyroid function test, and vitamin test (Figure 1). The phenotypes, with *P* < 0.05 in *t*-test and VIP>1 in OPLS-DA analysis, were defined as significantly variated clinical phenotypes related to GDM. Based on the criteria, 22 clinical phenotypes were significantly changed between GDM and control groups, with their *P* values shown in Figure 4.

The levels of C-reactive protein, cholinesterase, low-density lipoprotein (LDL), and triglyceride were significantly increased in the GDM group, which are recognized clinical indicators that significantly increase the risk of cardiovascular disease (Figure 4), while the high density lipoprotein (HDL), which is commonly known as “vascular scavenger,” was found to be significantly lower in the GDM pregnancy than the controls in the first trimester of pregnancy. The low serum HDL levels may also contribute to the risk of cardiovascular disease. Clinical indicators related to iron metabolism were found to be significantly changed between the GDM and the control groups, indicating that abnormal iron metabolism would occur in women with GDM in the first trimester (Figure 4). Inflammation-related indicators, including mean platelet volume, large platelet ratio, platelet volume distribution width, absolute value of neutrophils, leukocyte, platelet, and C-reactive protein, were all significantly different between GDM and control groups (Figure 4).

### 3.5. Defining of Potential Biomarkers for the Early Diagnosis of GDM

**Figure 5:**
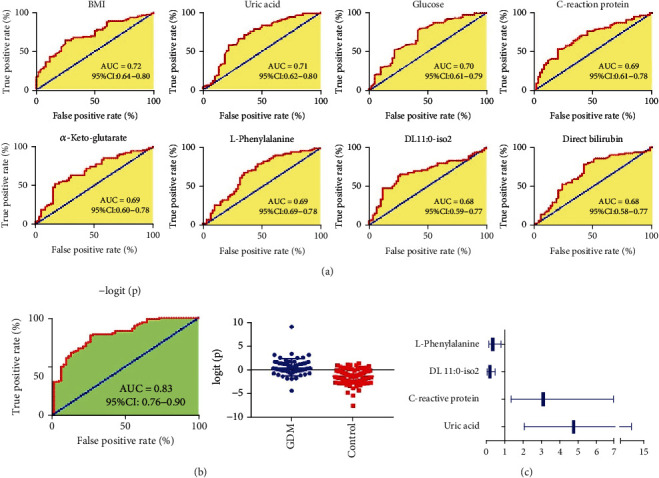
(a) Diagnostic performance of metabolic and clinical phenotypes for the discrimination of GDM women from healthy controls. (b) ROC curve and scatter plot of the phenotype panel. (c) A forest map showing the OR values and the corresponding 95% CI of the 4 most significantly changed phenotypes.

To investigate the predictive potential of these serum phenotypes in the first trimester for GDM, all the significantly changed phenotypes, including 36 metabolites and 22 clinical indicators, were submitted to ROC analysis. As shown in Figure 5(a), 8 phenotypes were found to the possessed area under the ROC curve (AUC) larger than 0.68, including BMI, uric acid, glucose, C-reaction protein, *α*-keto-glutarate, L-phenylalanine, DL 11 : 0-iso2, and direct bilirubin. Among these 8 phenotypes, the AUC value of pre-pregnancy BMI was the highest. Stratification of GDM population according to the pre-pregnancy BMI was shown in Figure S3. High pre-pregnancy BMI level has been widely proved to be associated with higher risk of GDM [6].

Subsequently, a binary logistic regression analysis and optimized algorithm of the backward stepwise (Wald) method were used to construct the optimal model for GDM prediction using these 8 potential phenotypes. Result showed statistically significant differences in glucose, uric acid, DL 11 : 0-iso2, L-phenylalanine, and direct bilirubin levels between GDM and controls even after adjusting for age.While the BMI, C-reaction protein, and*α*-keto-glutarate were not independent risk or protective factors for GDM. The phenotype panel for the early prediction of GDM was constructed as follows: logit(*p* = GDM) = 1.124 × [Glucose] + 0.0111 × [Uric acid] − 48.868 × [DL 11 : 0 − iso2] − 0.000754 × [L − phenylalanine] − 0.297 × [Direct bilirubin] − 1.454. In this equation, logit(*p* = GDM) is the predicted probability of GDM. A ROC curve and scatter plot based on the logit(*p* = GDM) values are shown in Figure 5(b). The phenotype panel showed a higher prediction performance for GDM than any of the individual phenotype, with AUC 0.83. All these results demonstrated the phenotype panel established from the serum metabolites and clinical indicators in the first trimester can be used for the early prediction of GDM with good sensitivity and specificity.

To further investigate whether these phenotypes are risk factors or protective factors for GDM, the concentrations values of uric acid, glucose, C-reaction protein, *α*-keto-glutarate, L-phenylalanine, DL 11 : 0-iso2, and direct bilirubin in each sample were all converted to ordered binary variables, with the minimum 50% defined as low level, while the maximum 50% defined as high level. The converted ordered binary variables, together with the BMI values, were used for binary logistic regression analysis. Odds ratio (OR) values and the corresponding 95% CI of the four most significantly changed phenotypes are shown in Figure 5(c) in the form of a forest map. High serum uric acid and C-reactive protein levels in early pregnancy were discovered to be the risk factors of GDM, with ORs 4.76 (95% CI: 2.08-10.90) and 3.10 (95% CI: 1.38-6.96), respectively.While high DL 11 : 0-iso2 and L-phenylalanine levels in early pregnancy were the protective factors of GDM, with ORs 0.18 (95% CI: 0.08-0.41) and 0.34 (95% CI: 0.15-0.76), respectively.

## 4. Discussion

In this study, perturbations of metabolites and clinical indicators related to GDM were comprehensively explored based on the results of targeted metabolomics analysis and clinical laboratory test results. A dozen of acyl carnitines were significantly lower in the GDM group compared to the control group (Figure 2(d)), while levels of fatty acids were the opposite of acyl carnitines. Acyl carnitines are generated by the combination of fatty acids and carnitine [27]. They are the intermediate products of fatty acid oxidation, facilitating the transporting of fatty acids to mitochondria [27, 28]. The down-regulation of acyl carnitines and up-regulation of fatty acids indicated that the oxidation level of fatty acids was reduced in the first trimester of GDM mothers. According to the previous literature report, high glucose levels reduced fatty acid oxidation and increased triglyceride accumulation in the placenta of GDM pregnant women [29]. They revealed an unrecognized regulatory mechanism on placental fatty acid metabolism by which high glucose levels reduced mitochondrial fatty acid oxidation of carnitine palmitoyl transferase І, shifting flux of fatty acid away from oxidation toward the esterification pathway, thus leading to the accumulation of placental triglycerides. In the present study, the metabolic phenotype variation related to GDM revealed the down-regulation of fatty acid oxidation even in the first trimester of GDM mothers. Actually, the decreased fatty acid oxidation may already present in GDM women before gestation. As shown in Table 1, significant higher pre-pregnancy BMI values were observed in the GDM women compared to the controls, indicating that the women with GDM have a higher rate of obesity before gestation. Evidence indicated that obesity causes a decrease in fatty acid oxidation, which contributes to lipid accumulation within the cells, conferring more susceptibility to cell dysfunction and increasing the risk of type 2 diabetes mellitus [30].

The content of pyruvate (Figure 2(d)), a product of glycolysis, was significantly higher in the GDM group than the control group. Approximately, 16-20% of plasma pyruvate stems from alanine, which results in a strong positive correlation between alanine and pyruvate (Figure 3) [31]. In this study, the serum level of alanine was significantly higher in the GDM women than the controls. The elevated alanine, a highly gluconeogenic amino acid, was proved to contribute to the glucose intolerance and insulin resistance [32].

Correlation analysis revealed that the acyl carnitines showed strong correlations with dehydroepiandrosterone (Figure 3(a)). Dehydroepiandrosterone is a natural steroid hormone produced from cholesterol by the adrenal glands [33]. Consolidated data showed that the dehydroepiandrosterone regulated the metabolism of lipid compounds [34–36]. Hepatocytes treated by dehydroepiandrosterone exhibited a decline in acyl carnitines, which reflected the decline in fatty acid catabolism [37]. The strong correlations existed between dehydroepiandrosterone and acyl carnitines indicated that dehydroepiandrosterone may regulate the oxidation of fatty acids by regulating the metabolism of acyl carnitine, thus regulating the whole lipid metabolism network. The low serum level of dehydroepiandrosterone in the first trimester of pregnancy (Figure 2(d)) might be one of the reasons why GDM mother has a higher risk of lipid metabolism disorder and cardiovascular disease. Besides the regulation of lipid metabolism, dehydroepiandrosterone was also proved to decrease hyperglycemia and increase insulin sensitivity [38, 39]. Administration of dehydroepiandrosterone can decrease the levels of hepatic gluconeogenic enzyme [39]. Cho et al. proved that dehydroepiandrosterone may suppress gluconeogenesis by increasing Akt phosphorylation [40].

On the other hand, the significantly variated clinical phenotypes mainly involved in the increased risk of cardiovascular disease, inflammation, and iron deficiency anemia. Actually, cardiovascular risk postpartum of GDM women has been widely proved and gained widespread attention. As reported in a previously published systematic review, the GDM women have a two-fold higher risk of cardiovascular events postpartum compared with their peers [5]. This risk is not dependent upon intercurrent type 2 diabetes and is apparent within the first decade after pregnancy. Thus, even without progressing to type 2 diabetes, women with GDM comprise an at-risk population for cardiovascular disease [5]. Studies have shown that platelets play an important role in the intercellular community, immunization, and inflammatory activity [41]. Previous literature reports showed that women with GDM had significantly higher values of platelet than healthy pregnant women in the second trimester [42]. In the present study, the high platelet level of GDM was observed in the first trimester of pregnancy. Besides the platelet indices, leukocyte, neutrophils, and C-reactive protein were also significantly higher in the GDM group than the control group, which was also consistent with the research results reported in the literature. All these indicated that pregnant women with GDM had higher levels of inflammation even in their early pregnancy.

Serum transferrin is essential for the regulation of iron metabolism. It is responsible for the transport of iron in a soluble, nontoxic form among different tissues and organs. In this study, serum transferrin levels were significantly higher in the diabetic group than in the control group. Both iron deficiency and hypoxia were proved to increase the serum levels of transferring [43–45]. Considering that there was no significant difference in serum iron content between the two groups, we hypothesized that hypoxia caused the up-regulation of transferrin in GDM women. Hypoxia increased the rate of erythropoiesis and also the level of circulating transferring [45]. Chronic intrauterine hypoxia caused by GDM was proved to be the most likely cause of stillbirths during the last weeks of pregnancy [46]. The high serum transferrin level suggested that hypoxia may occur in the first trimester of GDM mothers. Since transferrin is the main transport protein for iron, levels of total ion binding capacity can be estimated by multiplying the transferrin concentration by a converting factor. Consistent with transferrin, the total ion binding capacity also presented a significantly higher level in GDM women [44]. The other two indices, including iron saturation and transferrin saturation, are negatively correlated with transferring [44]. The negative relationships are given an explanation of the lower levels of iron saturation and transferrin saturation in GDM women.

Besides these findings above, several limitations also need to be addressed in this study. First, this study is a single-hospital center clinical study that only included Chinese pregnant women. Therefore, the predictive phenotype panel cannot be directly applied to the other GDM population or to other ethnic groups. Second, as a pilot study, the number of participants was relatively small. The predictive phenotype panel needs to be validated in larger sample sizes and in broader populations. Third, this study only looked at data from a single point in the first trimester and did not track changes of the predictive phenotype panel throughout the whole pregnancy.

## 5. Conclusions

In summary, biomarkers were explored for the early diagnosis of GDM in the first trimester. A phenotype panel was established for the early diagnosis of GDM based on the serum concentrations of glucose, uric acid, DL 11 : 0-iso2, L-phenylalanine, and direct bilirubin, with AUC 0.83 (95% CI: 0.76-0.90). Binary logistic regression analysis revealed that uric acid and C-reactive protein levels were the risk factors of GDM, while DL 11 : 0-iso2 and L-phenylalanine levels were the protective factors of GDM. The current findings suggested that women with GDM exhibited broad-scale perturbation of metabolic and clinical phenotypes even in the first trimester of pregnancy. Through further validation with a larger sample size, the phenotype panel established in this study is expected to be clinically used for the early warning and diagnosis of GDM in the first trimester of pregnancy.

## Figures and Tables

**Table 1:** Demographic and clinical characteristics of the GDM group and the control group.

Characteristic	GDM group	Control group	*P* value
Number (*n*)	60	90	
Maternal age (mean ± SD, years)	30.8 ± 4.0	30.4 ± 2.6	0.86
Embryonic age at collection (mean ± SD, weeks)	9.7 ± 1.8	10.2 ± 1.8	0.08
Nationality			0.42
Han	56 (93)∗	87 (97)	
Manchu	2 (3)	2 (2)	
Hui	1 (2)	0	
Ewenki	1 (2)	0	
Korean	0 (0)	1 (1)	
Pre-pregnancy BMI (mean ± SD, kg/m^2^)	25.1 ± 4.0	22.4 ± 4.1	<0.01
Low	0 (0)	8 (9)	
Normal	28 (47)	55 (61)	
Overweight	32 (53)	27 (30)	
Parity			0.59
Primipara	32 (53)	44 (49)	
Multiparas	28 (47)	46 (51)	
Family history of diabetes			0.14
Yes	11 (18)	9 (10)	
No	49 (82)	81 (90)	
Infant gender			0.64
Male	35 (58)	49 (54)	
Female	25 (42)	41 (46)	
Infant birth weight (mean ± SD, g)	3481.1 ± 617.7	3345.1 ± 514.2	0.03
Infant length (mean ± SD, cm)	50.0 ± 2.1	49.7 ± 1.4	0.07

^∗^Data are presented as mean ± SD or participant numbers (%), unless otherwise specified. *P* values were calculated by hypothesis testing. For continuous variables, the distribution of the variable was first assessed by the Shapiro-Wilk test. Bilateral Student's*t*-test was used for normally distributed data, while the Mann–Whitney*U*test was used for nonparametric data. For categorical variables, *P* values were calculated using the chi-square test.

## Data Availability

The metabolomics profiling data of this manuscript has been uploaded the Figshare (https://figshare.com) with doi (doi:10.6084/m9.figshare.13317242).

## Supplementary Materials

Supplementary Materials Figure S1. PCA analysis of the metabolomics data. Figure S2. Random permutation test of the OPLS-DA model with 100 iterations. Figure S3. Stratification of GDM population according to the pre-pregnancy BMI. Table S1. Information of the 36 significantly changed metabolites between control and GDM groups. Table S2. Significantly changed phenotypes between normal and overweight GDM women.
