# Supplementary material for: UHPLC-MS/MS-Based Metabolomics and Clinical Phenotypes Analysis Reveal Broad-Scale Perturbations in Early Pregnancy Related to Gestational Diabetes Mellitus
Source: Dis Markers. 2022 Aug 24;2022:4231031. doi: 10.1155/2022/4231031 (PMC9433254; doi:10.1155/2022/4231031)
Supplement: Supplementary Materials — Figure S1. PCA analysis of the metabolomics data. Figure S2. Random permutation test of the OPLS-DA model with 100 iterations. Figure S3. Stratification of GDM population according to the pre-pregnancy BMI. Table S1. Information of the 36 significantly changed metabolites between control and GDM groups. Table S2. Significantly changed phenotypes between normal and overweight GDM women. [file 4231031.f1.docx]

**UHPLC-MS/MS Based Metabolomics and Clinical Phenotypes Analysis Reveal Broad-Scale Perturbations in Early Pregnancy Related to Gestational Diabetes Mellitus**

Ting Hu ^a, #^, Zhuoling An ^a, #^, Han Li ^a^, Yanping Liu ^b^, Liangyu Xia ^b^, Ling Qiu ^b^, Aimin Yao ^c^, Liangkun Ma ^b,^ *, Lihong Liu ^a,^ *

*^a^ Beijing Chao-Yang Hospital, Capital Medical University, Beijing 100020, PR China*

*^b^ Peking Union Medical College Hospital，China Academic Medical Science and Peking Union Medical College, Beijing, 100730, PR China;*

*^c^ Shunyi District Maternal and Child Health Hospital, Beijing, 101320, P. R. China*

^#^Coauthors: Ting Hu and Zhuoling An contributed equally.

*Corresponding authors: Liangkun Ma and Lihong Liu.

*E-mail address*: gtnlcyyy@163.com (Prof. Liu); liangkun_ma@yahoo.com (Prof. Ma).

*Tel*.: +86-10-85231786

**Content:**

- Figure S1. PCA analysis of the metabolomics data.
- Figure S2. Random permutation test of the OPLS-DA model with 100 iterations.
- Figure S3. Stratification of GDM population according to the pre-pregnancy BMI.
- Table S1. Information of the 36 significantly changed metabolites between control and GDM groups.
- Table S2. Significantly changed phenotypes between normal and overweight GDM women

**Graphic abstract**

**Figure S1.** (A) PCA score plot based on the quantification data of the 184 metabolites. (B) PCA score plot based on the concentration levels of the 36 significantly changed metabolites .


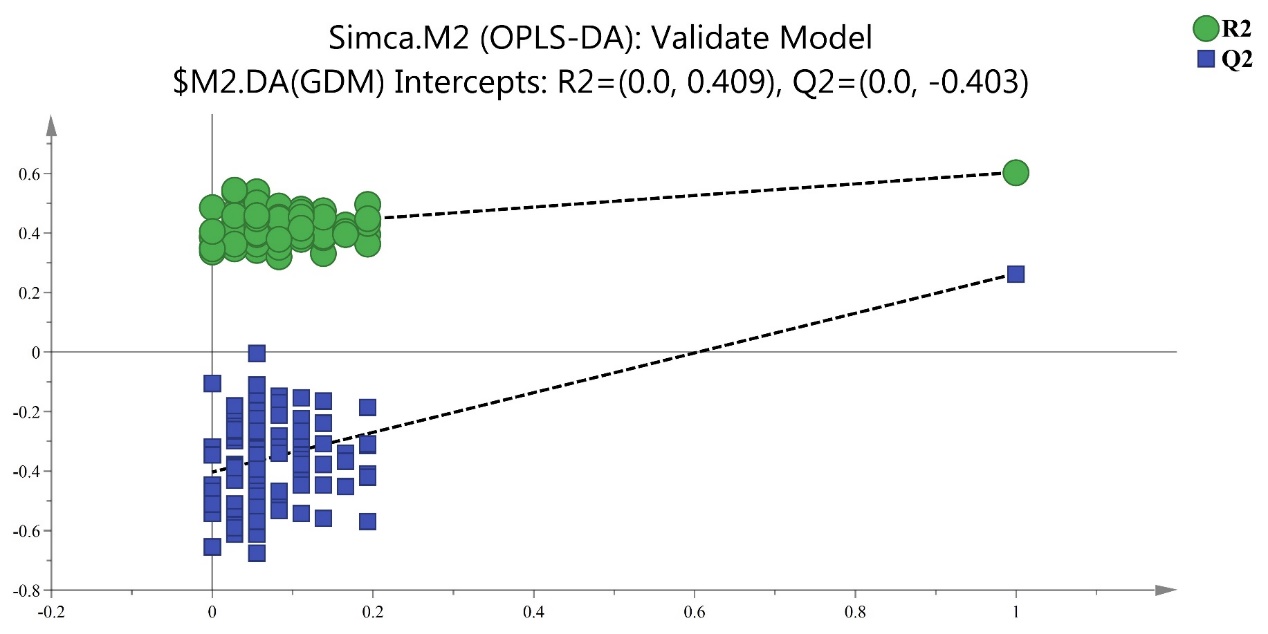


**Figure S2.** Random permutation test of the OPLS-DA model with 100 iterations.

**Figure S3.** Stratification of GDM population according to the pre-pregnancy BMI. (A) PCA score plot. (B) OPLS-DA score plot. (C) Random permutation test of the OPLS-DA model with 100 iterations.

**Table S1.** Information of the 36 significantly changed metabolites between control and GDM groups.

| **Metabolites** | **P value** | **FDR** | **VIP** | **Fold change** |
| --- | --- | --- | --- | --- |
| L-Phenylalanine | 0.0002 | 0.0196 | 2.17 | 0.91 |
| α-Keto-glutarate | 0.0002 | 0.0196 | 1.71 | 0.87 |
| DL 11:0-iso2 | 0.0004 | 0.0196 | 2.02 | 0.73 |
| DL 12:0-iso2 | 0.0004 | 0.0196 | 1.97 | 0.74 |
| L-Tryptophan | 0.0007 | 0.0257 | 1.93 | 0.93 |
| DL 14:1-iso2 | 0.0018 | 0.0507 | 1.85 | 0.77 |
| DL 10:0 | 0.0021 | 0.0507 | 1.98 | 0.70 |
| DL 14:2-iso1 | 0.0035 | 0.0592 | 1.78 | 0.78 |
| DL 14:1-iso1 | 0.0069 | 0.1062 | 1.73 | 0.80 |
| Glycine | 0.0093 | 0.1197 | 1.45 | 0.90 |
| DL 16:2 | 0.0116 | 0.1197 | 1.72 | 0.82 |
| DL 14:1-iso3 | 0.0124 | 0.1197 | 1.59 | 0.85 |
| DL 14:0 | 0.0134 | 0.1197 | 1.70 | 0.87 |
| Octanoyl-L-carnitine | 0.0137 | 0.1197 | 1.77 | 0.71 |
| Dehydroepiandrosterone | 0.0157 | 0.1260 | 1.77 | 0.71 |
| DL 8:1-iso3 | 0.0192 | 0.1410 | 1.57 | 0.85 |
| DL 10:1-iso1 | 0.0200 | 0.1410 | 1.66 | 0.78 |
| L-Serine | 0.0278 | 0.1586 | 1.56 | 0.94 |
| DL 16:1 | 0.0287 | 0.1586 | 1.57 | 0.88 |
| DL 6:1-iso2 | 0.0300 | 0.1586 | 1.38 | 0.85 |
| L-Asparagine | 0.0327 | 0.1674 | 1.34 | 0.94 |
| N,N-Dimethylglycine | 0.0342 | 0.1701 | 1.61 | 0.90 |
| DL 12:0-iso1 | 0.0406 | 0.1866 | 1.04 | 0.78 |
| DL 14:2-iso2 | 0.0433 | 0.1927 | 1.49 | 0.87 |
| DL 10:1-iso2 | 0.0440 | 0.1927 | 1.50 | 0.87 |
| Nordeoxycholic acid | 0.0302 | 0.1586 | 1.06 | 1.12 |
| Xanthine | 0.0296 | 0.1586 | 1.09 | 1.09 |
| FA 22:3-iso1 | 0.0207 | 0.1410 | 1.31 | 1.13 |
| FA 22:4 | 0.0143 | 0.1197 | 1.52 | 1.16 |
| Valerylcarnitine-iso2 | 0.0136 | 0.1197 | 1.67 | 1.21 |
| Pyruvate | 0.0128 | 0.1197 | 1.09 | 1.16 |
| Uracil | 0.0126 | 0.1197 | 1.05 | 1.08 |
| FA 20:3-iso1 | 0.0116 | 0.1197 | 1.48 | 1.17 |
| Xanthosine | 0.0028 | 0.0521 | 1.26 | 1.17 |
| L-Alanine | 0.0025 | 0.0507 | 1.07 | 1.10 |
| L-isoleucine | 0.0023 | 0.0507 | 1.00 | 1.08 |

**Table S2.** Significantly changed phenotypes between normal and overweight GDM women

| **Name** | **P value** | **VIP** |
| --- | --- | --- |
| Cholinesterase | 0.0004 | 2.19 |
| Uric acid | 0.0007 | 2.23 |
| Glutaconylcarnitine | 0.0018 | 1.80 |
| Glutenyl transaminase | 0.0023 | 1.71 |
| Absolute value of Eosinophil | 0.0029 | 1.52 |
| Red cell distribution width | 0.0071 | 1.45 |
| Eosinophil percentage | 0.0084 | 1.49 |
| 1-Methyluric acid | 0.0088 | 1.50 |
| DL 10:3-iso1 | 0.0104 | 1.58 |
| Urate | 0.0118 | 1.92 |
| Tetraiodothyraxine | 0.0165 | 1.32 |
| Lithocholic acid | 0.0168 | 1.10 |
| FA 22:4 | 0.0171 | 1.84 |
| LDL | 0.0178 | 1.51 |
| NG,NG-Dimethylarginine | 0.0187 | 1.27 |
| Neutrophils Percentage | 0.0193 | 1.25 |
| FA 16:0 | 0.0223 | 1.94 |
| N-Acetylaspartate | 0.0230 | 1.66 |
| Vitamin A | 0.0246 | 1.34 |
| α-Keto-glutarate | 0.0262 | 1.47 |
| FA 22:3-iso1 | 0.0275 | 1.76 |
| FA 20:3-iso1 | 0.0292 | 1.77 |
| FA 22:5- n3 | 0.0297 | 1.61 |
| FA 20:2 | 0.0300 | 1.68 |
| FA 16:1 | 0.0305 | 1.58 |
| Hippurate | 0.0319 | 1.39 |
| Total cholesterol | 0.0322 | 1.34 |
| Uridine | 0.0339 | 1.24 |
| Thyroid hormone | 0.0340 | 1.08 |
| Absolute value of Lymphocyte | 0.0354 | 1.38 |
| 7-Methylxanthine | 0.0360 | 1.00 |
| DL 8:1-iso1 | 0.0387 | 1.54 |
| FA 17:1 | 0.0390 | 1.61 |
| FA 18:0 | 0.0393 | 1.62 |
| FA 20:1-iso1 | 0.0396 | 1.54 |
| Lymphocyte percentage | 0.0415 | 1.20 |
| FA 17:0-iso2 | 0.0430 | 1.53 |
| Hyodeoxycholic acid-iso1 | 0.0477 | 1.50 |
| FA 18:2 | 0.0491 | 1.63 |
| DL 10:3-iso2 | 0.0492 | 1.39 |
